# Merging clinical research and standard healthcare – Nordic Precision Cancer Medicine Symposium 2025

**DOI:** 10.2340/1651-226X.2026.45168

**Published:** 2026-03-04

**Authors:** Elisa Bjørgo, Nina Ånensen, Gro Live Fagereng, Hege G. Russnes, Sigbjørn Smeland, Kjetil Taskén, Åslaug Helland

**Affiliations:** a Institute for Cancer Research, Division of Cancer Medicine, Oslo University Hospital, Oslo, Norway; b MATRIX, Norwegian Centre for Clinical Cancer Research, Division of Cancer Medicine, Oslo University Hospital, Oslo, Norway; c Division of Cancer Medicine, Oslo University Hospital, Oslo, Norway; d Institute of Clinical Medicine, University of Oslo, Oslo, Norway; e Department of Pathology, Oslo University Hospital, Oslo, Norway

**Keywords:** Precision cancer medicine, precision diagnostics, functional precision oncology, precision immune oncology, clinical trials, health economics and implementation

## Introduction

The second Acta Oncologica Nordic Precision Cancer Medicine Symposium (NPCM2025), held from September 15 to 17, 2025, in Oslo, Norway, focused on the intersection of clinical research and standard healthcare in precision oncology and was hosted by Oslo University Hospital and the Norwegian Centre for Clinical Cancer Research, MATRIX. This biannual event, part of the Acta Oncologica conference series, brought together 180 participants from altogether 15 countries to learn more about and discuss innovative approaches and practices in precision cancer diagnostics and treatment.

In the changing landscape of oncology, the integration of precision medicine into clinical practice represents a significant shift toward a more tailored approach to cancer care, driven by advanced molecular profiling, innovative clinical trials, and an expanding array of targeted therapies and treatment options. Comprehensive genomic profiling (CGP) provides a detailed analysis of a patient’s tumor DNA (and RNA) to uncover a wide array of genetic alterations. This information helps in selecting targeted therapies that are specifically designed to address the identified disruptions, thereby improving treatment efficacy and reducing unnecessary side effects. On the other hand, cellular phenotype, mirrored by comprehensive proteomics and functional analyses, assesses cancer cell responses to different drugs in controlled *in vitro* settings. The latter involves evaluating the effectiveness of specific therapies against cancer cells derived from the patient’s tumor. By examining the functional responses of these cells to various treatments, clinicians can identify the most effective therapeutic options tailored to the individual’s unique tumor biology. Together, CGP, proteomics and functional precision oncology create a synergistic framework for precision cancer medicine (PCM). While CGP and proteomics identify the genetic landscape of the tumor and host response, functional assays validate and refine treatment options based on the actual patient’s tumor cell responses. This multimodal approach enhances the likelihood of successful outcomes, allowing patients to receive more personalized and effective cancer treatments.

The implementation of PCM in standard healthcare is progressing but several hurdles remain. Key challenges include unequal access to advanced diagnostics, uncertainties regarding the real-world effectiveness of targeted treatments, and issues related to reimbursement and co-payment for patients. In addition, there is a need for enhanced education and training for healthcare providers to effectively interpret genomic data and integrate it into clinical practice. Overcoming these barriers requires coordinated efforts among healthcare providers, policymakers, and stakeholders to ensure that PCM can be effectively incorporated into routine clinical practice, ultimately enhancing patient outcomes and equity in cancer care.

The second Nordic Precision Cancer Medicine Symposium brought together experts from different areas important for PCM implementation into standard healthcare, and topics addressed during the conference included precision diagnostics, functional precision oncology, precision immune-oncology, clinical trials as well as PCM implementation. In addition, current Nordic cancer strategies and PCM was discussed. This special edition focusing on PCM, includes publications from altogether 13 speakers and poster presenters from the NPCM2025 conference.

## Keynote insights: Pioneering advances in precision oncology and therapeutics

At NPCM2025, three keynote speakers showcased the latest advancements in the field of PCM.

Edwin Cuppen from the Hartwig Medical Foundation provided an insightful overview of the diagnostic applications of whole genome sequencing (WGS) in oncology across the Netherlands. Currently, around 1,100 patients from 30 hospitals are screened annually. In June 2025, the Dutch healthcare authorities approved reimbursement for all patients who were in sufficient condition to undergo treatment. Although routine WGS-based cancer diagnostics can be beneficial for patient care, there are still significant challenges to widespread implementation. Key obstacles include the requirement for high-quality, non-formalin fixed paraffin embedded (FFPE) tissue samples and high sequencing costs. To enhance cancer care, a WGS database is being built at Hartwig to support research efforts, with new findings continually integrated into the database, thus refining cancer care practices over time. Finally, Cuppen discussed the potential for reusing data from this comprehensive database, which not only encompasses sequencing data but also includes clinical metadata. International data sharing is feasible, and fostering global collaborations will be crucial for optimizing the use of this and similar resources.

Andreas Bjerrum from Copenhagen University Hospital in Denmark emphasized the significance of secondary use of health data for advancing precision oncology. He pointed out that patients with the same diagnosis can respond differently to identical treatments, making it crucial to analyze real-world data (RWD) from diverse patient populations to identify potential patterns. By leveraging these insights, healthcare providers can better understand which patients are likely to benefit from specific therapies and which may not respond, ultimately enhancing patient care. To facilitate this process, data can be structured and harmonized using frameworks such as OMOP-CDM (Observational Medical Outcomes Partnership – Common Data Model), which enables federated analyses. The standardization and harmonization of RWD allows us to learn from every patient, providing a robust platform for advancing PCM. Bjerrum’s presentation on leveraging data from the Capital Region of Denmark exemplified how real-world applications of precision medicine can enhance patient outcomes through informed treatment choices. In this Acta Oncologica special edition, Bjerrum et al. commented on a qualitative study and highlighted the shared responsibility between the public and private sectors in supporting evidence generation for post-approval assessment of precision medicine [1].

Patricia LoRusso from the Yale Cancer Center presented an overview of antibody drug conjugates (ADCs) in cancer treatment, discussing both current challenges and new, innovative strategies. ADCs have transformed drug delivery, with 450 different ADCs entering clinical development by September 2025, 13 of which have received U.S. Food and Drug Administration (FDA) approval. Key barriers for enhancing ADC efficacy and reducing toxicity include limited payload diversity, linker stability, and the translation of results from preclinical models to humans. LoRusso highlighted the modular nature of ADCs and various optimization opportunities. The next-generation ADCs focus on overcoming the abovementioned barriers. Notable advancements include bispecific ADCs targeting two different antigens, potentially overcoming resistance and enhancing specificity. While many bispecific ADCs are in clinical development, most utilize the same payloads. However, dual-payload ADCs are now entering clinical development. In addition, combining ADCs with standard therapies shows substantial promise.

## Conference sessions: Shaping the future of precision cancer care

The conference consisted of four sessions, each exploring key areas of PCM: precision diagnostics, functional precision oncology and precision immune oncology, clinical trials and the implementation of PCM in standard healthcare. Each session featured three internationally renowned speakers who presented cutting-edge research in their respective fields. In addition, selected short talks from abstract submissions enriched the program.

Session one explored advances in precision diagnostics with insights from three invited speakers: Alona Sosinsky from Genomics England, Janne Lehtiö from Karolinska Institutet in Stockholm and Kushtrim Kryeziu from Oslo University Hospital. Sosinsky described ways to optimize genomic testing, presenting tools for variant interpretation and stressing the importance of translating new technologies into clinical practice. Lehtiö highlighted the critical role of proteomics within precision medicine, demonstrating its application in identifying cancer subtypes and refining patient stratification via proteogenomics. He advocated for a multimodal diagnostic approach, including MS-based proteomics, in clinical trials and to support routine clinical decision making, while noting the current lack of large cohort studies combining proteogenomics and clinical outcomes. Kryeziu discussed functional precision oncology, sharing insights from the EVIDENT trial that uses multimodal diagnostics for treating metastatic colorectal cancer (CRC). This includes *ex vivo* drug-sensitivity testing on patient-derived tumor organoids (PDOs) in addition to molecular profiling, both DNA, RNA and selected proteins, of tumor tissue as well as PDOs. To catch tumor heterogeneity, several PDOs are generated per tumor. Moreover, the large living CRC biobank enables discovery of drug and combination sensitivities in rare patient subgroups. In this NPCM2025 special edition, Welén et al. present SPRINTR (Swedish Prostate Cancer Initiative for Novel Treatment Regimens), a national research structure for better diagnosis and studies of prostate cancer [2].

Session two focused on functional precision oncology and precision immune oncology. Diana Azzam from Florida International University described how technological advancements and the growth in approved drugs are facilitating the integration of genomics with functional drug testing as clinical decision support tools, thereby enhancing patient care [3]. Her data showed improved clinical outcomes for several paediatric cancer patients guided by functional precision medicine. Thorsten Zenz from the Univeristy Hospital Zurich described how drug perturbation of primary cancer cells can elucidate pathway connectivity within tumors and capture the effects and functions of mutations. He introduced the innovative INTERCEPT project, targeting children and adults with aggressive blood cancers. The project aims to overcome treatment resistance through intercepting of clonal expansion. By analyzing tumor material together with healthy blood cells, researchers can create a detailed map of therapy response at the single-cell level. This approach enhances the understanding of how tumor and healthy immune cells interact and respond to various treatments. Heidi Haikala from the University of Helsinki delivered a talk on precision immune oncology with a focus on lung cancer. She discussed the development of complex patient-derived organoids and tumor-on-chip models, which incorporate various cell types. These innovative systems replicate the complexity of cancer seen in patients, enabling researchers to uncover new mechanisms of drug resistance and response. Furthermore, they facilitate the testing of therapeutic interventions in a context relevant to patient care.

Session three highlighted the necessity for innovative clinical trials in the field of PCM. Anna Martling from the Karolinska Institutet in Stockholm presented findings from the Nordic ALASCCA trial [4]. This biomarker-driven, double-blinded and randomized controlled trial (RCT) included patients with primary rectal or colon cancer who had somatic alterations in PI3K pathway genes. Participants received either 160 mg of aspirin or a placebo daily as adjuvant therapy after surgery for 3 years. The ALASCCA trial is the first RCT to demonstrate that aspirin significantly reduces the recurrence rate in PI3K-altered primary CRC. This finding has the potential to change clinical practice for approximately one-third of patients with early CRC by repurposing a safe, inexpensive and globally accessible drug. Short talk speaker Sheraz Yaqub presented findings from the ASAC trial, which examined the role of aspirin as a secondary prevention for CRC liver metastases. The data revealed that adjuvant treatment with 160 mg aspirin over 3 years does not reduce the recurrence rate in this patient group. Thus, the protective effect of aspirin observed in CRC is not transferable to metastatic disease. In this special edition, Yaqub and colleagues report outcomes after resection of distal cholangiocarcinoma in a European patient cohort [5]. Ruth Plummer from Newcastle University provided insights into the academic drug discovery of rucaparib from a clinician’s perspective. Developed in collaboration with Cancer Research UK (CRUK) and Agouron-Pfizer, rucaparib is a poly (ADP-ribose) polymerase) (PARP) inhibitor that impedes cancer cells’ DNA repair mechanisms and is primarily used for patients with BReast CAncer gene (BRCA) mutations. Clinical trials, initiated in Newcastle in 2003, led to FDA and European Medicines Agency approvals in 2016 and 2018, respectively. Plummer highlighted that the success of the CRUK Newcastle Drug Discovery Unit is largely due to industry partnerships that facilitate the rapid transition of early drug projects to be progressed from the laboratory to the clinic. Kimmo Porkka from the University of Helsinki and Helsinki University Hospital emphasized the importance of cancer drug repurposing in precision cancer care. He found that relying solely on genomics for repurposing can be challenging, as many mutations are not actionable. In addition, it is difficult to predict treatment responses for patients with an actionable mutation. Porkka advocated for integrating functional and omics platforms to assess drug effects on patients’ cells in real-time. He emphasized the necessity of large-scale studies and close collaboration with the pharmaceutical industry, highlighting the advantages of European and global personalized medicine trials, such as the PRIME-ROSE network of DRUP-like clinical trials [6], for data generation and evidence building. In addition, he discussed the need to capture drug responses beyond clinical trials and described federated clinical data networks such as FinOMOP, VALO and DARWIN, which are designed for the generation and analysis of RWD. In this special edition, van der Pol et al. [7] and Augudo et al. [8] described data merging procedures in PRIME-ROSE. Furthermore, Slørdahl et al. described clinical outcomes following genomically guided trametinib monotherapy across cancer types in the IMPRESS-Norway trial [9]. Abel et al. introduced the new Swedish DRUP-like clinical trial, FOCU.SE [10], and Tryggvadottir et al. presented data on targeted therapy for lung cancer in Iceland [11]. Sommervoll et al. presented patient-reported quality of life data from a sub-study of the DART trial for patients with locally advanced non-small-cell lung cancer [12].

Session four centred on the implementation of PCM within standard healthcare systems. Christine Leopold from the Utrecht University presented the WHO Regional Office for Europe’s framework for use of managed entry agreements (MEA), providing practical checklists and advice. Moreover, she highlighted the clinician-initiated Drug Access Protocol (DAP) [13] platform in the Netherlands, which links evidence generation and reimbursement for precision cancer treatments. Leopold stressed the necessity of a collaborative platform that integrates clinicians, health technology assessment (HTA) bodies, payers and industry for effective implementation, and in this special edition, Leopold et al. described the stakeholders’ experiences with DAP [14]. David Thomson from the National Institute for Health and Care Excellence (NICE) in the UK shared insights from his experience with outcome-based MEA, advocating for the use of straightforward, hard endpoints, such as overall survival, to streamline these frameworks. As global spending on cancer medications continues to rise, payers are likely to implement policies and develop mechanisms to manage this increasing expenditure. Per-Olof Thuresson from Roche introduced the concept of data transportability for RWD, emphasizing the use of foreign data to inform decision-making. He highlighted the industry’s need for seamless data updates and predictability in the European data-sharing initiatives. In this Acta Oncologica special edition, short talk speaker Oskar Frisell from the Swedish Institute for Health Economics and coworkers presented a conceptual health economic modelling framework to assess the cost-effectiveness of molecular target driven treatment regimens in oncology [15].

## Cancer strategies in the Nordics

Cancer care and research in the Nordic countries are characterized by a collaborative and comprehensive approach to tackling cancer. Denmark, Norway and Sweden have recently launched updated national cancer strategies, while Finland released its first national cancer plan in November 2025.

Mef Nilbert from Lund University emphasized that key elements important for precision medicine have been incorporated into the updated Swedish cancer strategy, including the need for a national diagnostic network to ensure efficient services and equity of care. Additional initiatives include a pilot program for nationally coordinated implementation of advanced and innovative analyses, molecular tumor boards (MTBs) and formation of a precision medicine trial network. Recently, Sweden launched the national clinical trial FOCU.SE [10], a DRUP-like clinical trial that has received financial backing from the Swedish government. Ole Alexander Opdalshei from the Norwegian Cancer Society presented key elements of Norway’s new cancer strategy, which will guide initiatives over the next decade. Inspired by Europe’s Beating Cancer Plan, this ambitious strategy emphasizes cancer prevention, early detection, and access for all patients to comprehensive cancer centers and world-class cancer research. A notable goal of the strategy is to ensure that all cancer patients are offered genomic profiling when relevant to their treatment decisions. In addition, the national strategy for personalized medicine aims to make personalized medicine an integral part of the health service. Kimmo Porkka highlighted that Finland’s first national cancer strategy will be launched in late 2025, covering the coming decade. An accompanying action plan is scheduled for publication in 2026. Key focus areas include prevention and early detection, equitable and effective cancer care and adaptability within an evolving healthcare landscape. This includes plans for a Finnish Health Data Space. Denmark, in conjunction with its fifth cancer strategy launched in 2025, also introduced a new strategy for personalized medicine. Ulrik Lassen from Copenhagen University Hospital focused on the implementation of a fast-track approval system for clinical trials, which will reduce the assessment time for Phase I and Phase I/II trials to just 2 weeks starting in August 2025. Taken together, the Nordic countries are prioritizing cohesion, MTBs, the integration of genetic testing in earlier lines of treatment, the use of RWD and expedited approvals for clinical trials as they advance their cancer care and research initiatives.

## Conclusion

The second Nordic Precision Cancer Medicine Symposium brought together internationally renowned speakers and facilitated enhanced international collaboration. The presentations sparked engaging discussions, creating a vibrant and interactive atmosphere.

Looking ahead, there is a pressing need for more multimodal diagnostic approaches, clinical trials and collaborative international initiatives, including treatment of patients across borders. Additionally, a significant focus area will be the secondary use of data, where standardization, harmonization and federated analyses will be vital. Furthermore, international collaboration is essential for addressing and harmonizing legal frameworks to support these efforts.

## References

[1] Bjerrum A, Fanø A, Lassen U. Realizing precision oncology through shared real-world data infrastructure. Acta Oncol. 2026;65:156–58. 10.2340/1651-226X.2026.45074 41725268 PMC12936906

[2] Welén K, Ahlberg M, Akre O, Aly M, Andersson J, Andersson P, et al. SPRINTR: Swedish PRecision medicine Initiative for Novel Treatments and Research – towards efficient recruitment to clinical trials for prostate cancer. Acta Oncol. 2026;65. 10.2340/ao.v65.45126 PMC13151910 42080506

[3] Acanda De La Rocha AM, Berlow NE, Fader M, Coats ER, Saghira C, Espinal PS, et al. Feasibility of functional precision medicine for guiding treatment of relapsed or refractory pediatric cancers. Nat Med. 2024;30:990–1000. 10.1038/s41591-024-02848-4 38605166 PMC11031400

[4] Martling A, Myrberg IH, Nilbert M, Grönberg H, Granath F, Eklund M, et al. Low-dose aspirin for PI3K-altered localized colorectal cancer. N Eng J Med. 2025;393:1051–64. 10.1056/NEJMoa2504650 40961426

[5] Holm MB, Busund S, Rihel R, Sahakyan MA, Gladhaug IP, Verbeke CS, et al. Outcome of resectable distal cholangiocarcinoma in a European patient cohort: comparison of the 7th and 8th edition of the UICC/AJCC TNM classification. Acta Oncol. 2026;65:148–55. 10.2340/1651-226X.2026.44965 41711495 PMC12931082

[6] Taskén K, Haj Mohammad SF, Fagereng GL, Falk RS, Helland Å, van Waalwijk van Doorn-Khosrovani SB, et al. PCM4EU and PRIME-ROSE: collaboration for implementation of precision cancer medicine in Europe. Acta Oncol. 2024;63:385–91. 10.2340/1651-226X.2024.34791 38779910 PMC11332530

[7] van der Pol H, Kringelbach T, Agudo MM, Stav GB, Fagereng GL, Fiocco M, et al. Procedures of data merging in precision cancer medicine: the PRIME-ROSE Project. Acta Oncol. 2026;65:1–8. 10.2340/1651-226X.2026.44889 41496458 PMC12789942

[8] Agudo MM, van der Pol H, Stav GB, Kringelback T, Puco K, Flobak Å, et al. ‘Crossing borders’ in data standardisation: application of OMOP CDM in an international clinical trial network in precision cancer medicine. Acta Oncol. 2026;65:159–63. 10.2340/1651-226X.2026.45120 41733536 PMC12946775

[9] Slørdahl KS, Puco K, Falk RS, Dyvik I, Brabrand S, Niehusmann P, et al. Clinical outcomes of genomically guided trametinib monotherapy across cancer types: results from the IMPRESS-Norway trial. Acta Oncol. 2026;65:90–6. 10.2340/1651-226X.2026.45086 41664938 PMC12902912

[10] Abel E, Östling P, Hult EH, Kulbacka K, Babacic H, Baan A, et al. The FOCU.SE Trial: a nationwide swedish drug repurposing protocol and research framework. Acta Oncol. 2026. 10.2340/ao.v65.45355 PMC13081547 41972845

[11] Haraldsdottir S., Hilmarsdottir B, Barkardottir RB, Birgisson H, Gunnarsson Ö, Guðbjartsson T, et al. Targeted therapy in the treatment of lung cancer in Iceland 2010–2023. Acta Oncol. 2026. 10.2340/ao.v65.45118 PMC13071797 41954526

[12] Sommervoll FÅ, Horndalsveen H, Sommervoll DE, Koivunen J, Halvorsen TO, Grønberg BH, et al. How does pulmonary function impact QoL in patients with locally advanced NSCLC treated with chemoradiotherapy and durvalumab. Acta Oncol. 2026. 10.2340/1651-226X.2026.45040 PMC13006962 41854264

[13] Zeverijn LJ, van Waalwijk van Doorn-Khosrovani SB, Pisters van Roy AAMG, Timmers L, Tran THL, de Boer JE, et al. Harmonising patient-access programmes: the Dutch DRUG Access Protocol platform. Lancet Oncol. 2022;23:198–201. 10.1016/S1470-2045(21)00707-5 35114116

[14] Leopold C, Huisman AH, Vlaar KJGM, Bloemendal HJ, van Waalwijk van Doorn-Khosrovani SB. Stakeholders’ experiences with a clinician-imitated access program linking evidence generation and reimbursement for precision cancer treatments: the Drug Access Protocol in the Netherlands. Acta Oncol. 2026;65:22–31. 10.2340/1651-226X.2026.45000 41532708 PMC12816994

[15] Frisell O, Aas E, Henkel PS, Fagereng GL, Taskén K, Hult EH, et al. A conceptual health economic modelling framework to assess the cost-effectiveness of molecular target-driven treatment regimens in oncology. Acta Oncol. 2026;65:141–7. 10.2340/1651-226X.2026.45088 41711494 PMC12930514

